# Da-Bu-Yin-Wan Improves the Ameliorative Effect of DJ-1 on Mitochondrial Dysfunction Through Augmenting the Akt Phosphorylation in a Cellular Model of Parkinson’s Disease

**DOI:** 10.3389/fphar.2018.01206

**Published:** 2018-10-18

**Authors:** Yi Zhang, Xiao-Gang Gong, Hong-Mei Sun, Zhen-Yu Guo, Jing-Hong Hu, Yuan-Yuan Wang, Wan-Di Feng, Lin Li, Ping Li, Zhen-Zhen Wang, Nai-Hong Chen

**Affiliations:** ^1^Department of Anatomy, School of Traditional Chinese Medicine, Beijing University of Chinese Medicine, Beijing, China; ^2^College of Special Education, Beijing Union University, Beijing, China; ^3^Center for Scientific Research, School of Traditional Chinese Medicine, Beijing University of Chinese Medicine, Beijing, China; ^4^Key Laboratory for Neurodegenerative Diseases of Ministry of Education, Capital Medical University, Beijing, China; ^5^Beijing Key Lab for Immune-Mediated Inflammatory Diseases, Institute of Clinical Medical Science, China-Japan Friendship Hospital, Beijing, China; ^6^State Key Laboratory of Bioactive Substances and Functions of Natural Medicines, Neuroscience Center, Institute of Materia Medica, Chinese Academy of Medical Sciences, Peking Union Medical College, Beijing, China; ^7^College of Pharmacy, Hunan University of Chinese Medicine, Changsha, China

**Keywords:** Da-Bu-Yin-Wan, Parkinson’s disease, DJ-1, Akt, mitochondrial function

## Abstract

Da-Bu-Yin-Wan (DBYW) is recorded originally in China over six centuries ago, and it is used to treat Parkinson’s disease (PD) clinically in recent decades. DJ-1 is a homodimeric protein linked to early-onset PD, and found in the mitochondria. In addition, DJ-1 could protect the cells by regulating gene transcription and modulating the Akt signal pathways. Therefore, in this research, we aimed to investigate the ameliorative effect of DBYW on mitochondria in the view of the DJ-1 and Akt signaling. Rat adrenal pheochromocytoma cell line PC-12 was transfected with the plasmid pcDNA3-Flag-DJ-1 (pDJ-1). Subsequently, PC-12 cells were exposed to the PD-related mitochondrial toxin (1-methyl-4-phenylpyridinium) without/with the DBYW. After transfected with the plasmid pDJ-1, the 1-methyl-4-phenylpyridinium-induced toxicity was decreased, and the DJ-1 expression in protein level was increased. DJ-1 overexpression not only increased the mitochondrial mass, but also improved the total ATP content. Moreover, Akt phosphorylation was augmented by DJ-1 overexpression. Additionally, DBYW enhanced the above effects. Conclusively, these findings indicate that DBYW promotes the ameliorative effects of DJ-1 on mitochondrial dysfunction at least through augmenting the Akt phosphorylation in 1-methyl-4-phenylpyridinium-treated PC-12 cells.

## Introduction

Parkinson’s disease (PD) is a highly debilitating neurodegenerative disorder that induces body rigidity, tremor, bradykinesia, and postural instability (Kalia and Lang, 2015). The PD pathology is characterized by gradual loss of dopaminergic neurons in the substantia nigra (Pagonabarraga et al., 2015), the underlying mechanisms still need to be clarified even though the disease was first described 200 years ago (Przedborski, 2017). Compelling evidence from molecular studies and experimental animal models has demonstrated that mitochondrial dysfunction was associated with the pathogenesis of PD (Mattson et al., 2008). Given the critical role of mitochondria in cellular function, it is convincing that mitochondrial dysfunction has appeared as an important mechanism at the coincidence of genetic, environmental and neurotoxin threatens to PD (Dagda et al., 2009).

DJ-1, namely PARK7 (Parkinson protein 7), is a homodimeric protein highly conserved in divergent organisms and linked to early-onset PD (Bonifati et al., 2003). DJ-1 is detected in both the nucleus and cytoplasm, and found in the mitochondria (Zhang et al., 2005; Junn et al., 2009). DJ-1 could prevent the fragmentation of mitochondria (Blackinton et al., 2009), while DJ-1 mutations damage mitochondrial dynamics and lead to mitochondrial dysfunction (Wang et al., 2012). In addition, DJ-1 could protect the cells by regulating gene transcription and modulating cell signal pathways, e.g., Akt signaling (Wilson, 2011). Akt, a downstream protein of phosphoinositide 3-kinase (PI3K), is the essential mediator of neuron survival (Dudek et al., 1997). Akt exerts its neuroprotective effect on neuronal cells by phosphorylation (Franke et al., 2003), whereas Akt signaling defection has partly linked to the pathological process of PD (Burke, 2007; Levy et al., 2009). In addition, DJ-1 is important for Akt phosphorylation enhancement on oxidative stress in the models of PD (Aleyasin et al., 2010).

Da-Bu-Yin-Wan (DBYW) was originally interpreted in a traditional Chinese medicine (TCM) monograph *Dan Xi Xin Fa* authored by Dan-Xi Zhu, an outstanding TCM professionalist and physician during China Yuan Dynasty. DBYW is also recorded in the updated edition of *Pharmacopeia of People’s Republic of China* issued in the year of 2015 (Chinese Pharmacopoeia Commission, 2015). In China Ming Dynasty, Yi-Kui Sun (A.D. 1522–1619) firstly defined the disease dominated by body tremor as “Tremor Disease” in his literature *Chi Shui Xuan Zhu*. He considered by TCM theory that the tremor syndrome in the aged people resulted from multiple deficiencies in the human body, e.g., low Yin essence (Zhang et al., 2006). Accordingly, DBYW was employed as a TCM intervention to treat PD clinically in recent decades (Jia et al., 2010). Our previous studies demonstrate that DBYW increases the expression of tyrosine hydroxylase (TH) in SN, induces the ultrastructure change, and raises the level of monoamine neurotransmitters in the mice model of PD (He et al., 2010; Zhang et al., 2013). In addition, DBYW lessens the DNA damage of mitochondria, and increases the mitochondrial subunit NADH dehydrogenase 1 expression (Zhang et al., 2013). Moreover, DBYW up-regulates cellular adenosine 5′-triphosphate (ATP) content in the midbrain, and decreases the expression of ATP-sensitive potassium channel subunit (Gong et al., 2014). Additionally, DBYW could reduce the mitochondrial fragmentation induced by the PD-related mitochondrial toxin (1-methyl-4-phenylpyridinium) in human derived neuroblastoma cell line (Ma et al., 2015). However, the cellular mechanisms by which DBYW exerts its protective effect on mitochondria are not totally interpreted. Therefore, in this research, we examined the possible link between DBYW and mitochondria from DJ-1 and Akt signaling in the cellular model of PD.

## Materials and Methods

### Chemical Reagents and Antibodies

All reference standard chemicals were obtained from National Institutes for Food and Drug Control, China^1^, including berberine hydrochloride (C_20_H_18_ClNO_4_, PubChem CID: 12456, Lot No.: 111895–201504), mangiferin (C_19_ H_18_O_11_, PubChem CID: 5281647, Lot No.: 111607–201503), and phellodendrine chloride (C_20_H_24_ClNO_4_, PubChem CID: 59818, Lot No.: 110713–201212). Lipofectamine 2000 and MitoTracker Green (MTG) were purchased from Invitrogen (Grand Island, NY, United States). 1-methyl-4-phenylpyridinium (MPP^+^) were obtained from Sigma-Aldrich (St. Louis, MO, United States). The bicinchoninic acid kit, protease and phosphatase inhibitors, and enhanced chemiluminescence kit were bought from Applygen (Beijing, China). The used antibodies as the following: rabbit anti-DJ-1, rabbit anti-PI3K, rabbit anti-Akt, rabbit anti-Akt phosphorylation^Thr308^, rabbit anti-Akt phosphorylation^Ser473^ were obtained from Cell Signaling (Beverly, MA, United States). Mouse anti-beta-action primary antibody and all secondary antibodies were obtained from Zhong-Shan (Beijing, China).

### Preparation and Analysis for the Decoction

All components of DBYW were listed as the following (with Pharmacopoeia and local names) and described previously (Zhang et al., 2013, 2016a): Amur corktree bark (*Phellodendron chinense Cortex*; Huang-Bai) 12 g, Common anemarrhena rhizome (*Anemarrhenae Rhizoma*; Zhi-Mu) 12 g, Prepared rehmannia root (*Radix Rehmanniae Praeparata*; Shu-Di-Huang) 18 g, and Tortoise shell (*Carapax et Plastrum Testudinis*; Gui-Jia) 18 g. All components were obtained from Beijing Tong-Ren-Tang Nature Pharmacy (Beijing, China) and authenticated by the pharmacognosy professionals in the pharmacy. Briefly, the preparation method for the decoction was described previously (Chan et al., 2012). After treatment, final dose of the decoction extract was condensed to 1 g/ml (equivalent to dry weight of the component raw materials) by water bath. The extract was passed through a 0.22 μm filter (Millipore, Billerica, MA, United States), then divided and stored as the stock solution at -70°C.

Identification and quantification of the marker compounds in DBYW decoction were performed according to the method described in the updated *Pharmacopeia of People’s Republic of China* (Chinese Pharmacopoeia Commission, 2015), with minor modifications relating to the instrument and chromatographic conditions. Briefly, the marker compounds were analyzed using high performance liquid chromatography (Agilent 1100) with the diode-array detection (HPLC-DAD, Agilent, Santa Clara, CA, United States), respectively. Chromatographic separations were carried out using a Diamonsil C18 column (250 mm × 4.6 mm, 5-μm particle size, Dikma, Beijing, China), and appropriate mixture of acetonitrile/phosphoric acid /HPLC-grade water as the mobile phase. The mobile phase was filtered through a membrane (0.45-μm pore size) and degassed by ultrasonication before use. All measurements were made at a flow-rate of 1 mL/min, and detector was set for various compounds at different wavelength according to the China Pharmacopeia, respectively. The injection volume was 1 μL with analyte concentrations of 10–100 μg/mL, respectively. Analyte concentrations were adjusted to avoid overload of the columns. The integration of the chromatograms was performed with the Clarity software (Version 2.6.3, DataApex, Prague, Czechia). Peak areas in the chromatograms of DBYW were quantitated by external standard technique using solutions of the relative reference standards as described previously (Zhang et al., 2013).

### Cell Line and Culture Conditions

Rat PC-12 cells (adrenal gland, pheochromocytoma) were obtained from Institute of Materia Medica, Chinese Academy of Medical Sciences and Peking Union Medical College. PC-12 cells were grown in a culture mixture of Dulbecco’s modified Eagle’s medium (HyClone, Logan, UT, United States) containing 6% horse serum (Invitrogen, Grand Island, NY, United States) and 6% fetal bovine serum (Sijiqing, Hangzhou, China), supplemented with 1% streptomycin/penicillin (Gibco, Grand Island, NY, United States), in 5% CO_2_ humidified chamber at 37°C. The culture procedures were in strict compliance with proper cell density for all the following experiments.

### Transient Transfection and Treatments

**FIGURE 1:**
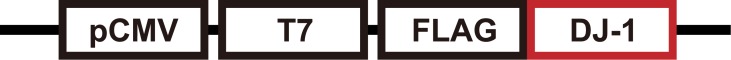
The plasmid pcDNA3-Flag-DJ-1 simplified structure.

The simplified structure of plasmid for expression of DJ-1 as described previously (Zhang et al., 2016b), pcDNA3-Flag-DJ-1 (pDJ-1), is displayed in **Figure 1**. The plasmid was validated by DNA sequencing and purified by the GoldHi plasmid kit (CoWin, Beijing, China) to remove endotoxin contamination. Cells were seeded at a density of 8 × 10^4^/well 24 h prior to transfection. For each well in 6-well plate, cells were transfected with pDJ-1 by the polycationic liposome-mediated transfection method, using the optimum amount of Lipofectamine 2000. Twenty-four hours post transfection, cells were exposed to the medium containing MPP^+^ (1 mM) with/without different doses of DBYW for 48 h, respectively. Experimental treatments are shown in **Table 1**.

**Table 1:** Experimental groups and treatments.

Groups	pDJ-1 transfection	MPP^+^ (1 mM)	DBYW (μg/ml)
Control	–	–	–
Model	–	+	–
Overexpression of DJ-1	+	+	–
DBYW at low concentration	+	+	20
DBYW at medium concentration	+	+	100
DBYW at high concentration	+	+	500


*DBYW, Da-Bu-Yin-Wan; MPP^+^, 1-methyl-4-phenylpyridinium.*

### Cell Viability Determination

Cell viability was assessed by using the Cell Counting Kit-8 (CCK-8) colorimetric assay (Dojindo, Kumamoto, Japan) (Ishiyama et al., 1997). Briefly, 24 h after previous cell transfection, PC-12 cells were seeded at a density of 8 × 10^4^ cells/mL in a 96-well plate and incubated for 24 h. Then various concentrations of DBYW were added with or without MPP^+^ (final concentrations mentioned in **Table 1**), respectively. Cells were incubated for a further 24 h, 10 μl of CCK-8 reagent was added to each well in a 96-well plate. After 1.5 h of incubation at 37°C, the absorbance was measured at a wavelength of 450 nm using the Safire2 microplate reader (Tecan, Männedorf, Switzerland). All results were expressed as compared to the control, which was defined as the baseline (100%).

### Western Blot Analysis

Cells were washed with phosphate-buffered saline solution, followed by lysis with radioimmunoprecipitation assay buffer containing protease and phosphatase inhibitors. The extract of total protein was run and separate on sodium dodecyl sulfate polyacrylamide gel electrophoresis, then transferred onto polyvinylidene difluoride membrane (Millipore, Billerica, MA, United States). Different blots were incubated overnight with primary antibodies against DJ-1 (1:1000 dilution), PI3K (1:1000), Akt (1:1000), β-action (1:2000), Phospho-Akt (Thr308) (1:500), Phospho-Akt (Ser473) (1:500), respectively; followed by horseradish peroxidase-conjugated secondary antibodies (1:2000) for 1 h. Then complexes were visualized with enhanced chemiluminescence kit. Signals on the Flims were quantified by densitometry performed with the Bio-Rad Quantity One software, Version 4.62 (Hercules, CA, United States). Beta-actin served as an internal control for DJ-1, PI3K, and Akt, respectively; whereas the total Akt as loading control for the Akt phosphorylation.

### Confocal Fluorescence Microscopy

To assess the mitochondrial mass, mitochondrial labeling was carried out using a cell-permeable fluorescent dye (MTG) based on the activity of mitochondria and involves minimal manipulation (Pendergrass et al., 2004). For visualization of mitochondria, cells were primarily treated with MTG (100 nM) for 15 min. Fluorescence was detected (490 nm/516 nm) by the confocal microscope FV1000 with the software Olympus FluoView Viewer, Version 3.1.2 (Olympus, Tokyo, Japan). Digital pictures were processed with the Image-Pro Plus software, Version 6.0 (Media Cybernetics, Bethesda, MD, United States).

### Total ATP Content Detection

Total ATP content was detected by the Stay Brite ATP bioluminescence assay kit (BioVision, Milpitas, CA, United States) according to the manufacturer’s protocol, based on the measurement for the firefly luciferase bioluminescence (Crouch et al., 1993). Briefly, PC-12 cells were subcultured in 96-well plates at a density of 8 × 10^4^ cells/ml. Twenty-four hours after various concentrations of DBYW treatment with or without MPP^+^ (1 mM), respectively. Then, 100 μl of ATP detection working solution was added to each well and incubated for 1 h at room temperature after lysed from the cells in the lysate buffer. The mixtures were centrifuged at 12,000 *g* for 30 s. The luminescence in the supernatant was recorded according to ATP-dependent luciferase activity, using the microplate reader Safire2 (Tecan, Männedorf, Switzerland). The bioluminescence value was normalized by the protein concentration that measured using bicinchoninic acid kit (Gong et al., 2014).

### Statistical Analysis

All result data are expressed as the mean ± standard deviation. Statistically significant differences among means were determined by one-way analysis of variance followed by Newman–Keuls’ *post hoc* tests, using the GraphPad Prism software, Version 6.02 (GraphPad, San Diego, CA, United States). A typical level at which the threshold of *P*-value is taken at 0.05.

## Results

### Analysis of Marker Compounds of DBYW

**FIGURE 2:**
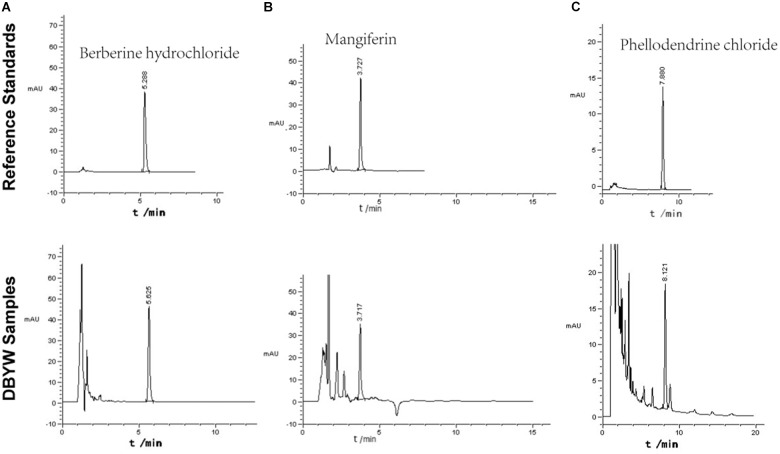
HPLC-DAD analysis of the DBYW decoction. Three reference standards were used for identifying and quantifying the marker compounds for DBYW. Panel **(A)** for berberine hydrochloride, panel **(B)** for mangiferin, and panel **(C)** for phellodendrine chloride.

The marker compounds in DBYW were analyzed with HPLC-DAD. By referring to reference standard chemicals, HPLC-DAD analysis indicated that the decoction contained the following marker compounds (*n* = 3): berberine hydrochloride (1.760 ± 0.033 mg/mL), mangiferin (0.501 ± 0.009 mg/mL), and phellodendrine chloride (0.476 ± 0.011 mg/mL). Chromatograms of the DBYW analyzed with relative reference standards are shown in **Figure 2**.

### DBYW Affects the Cell Viability

**FIGURE 3:**
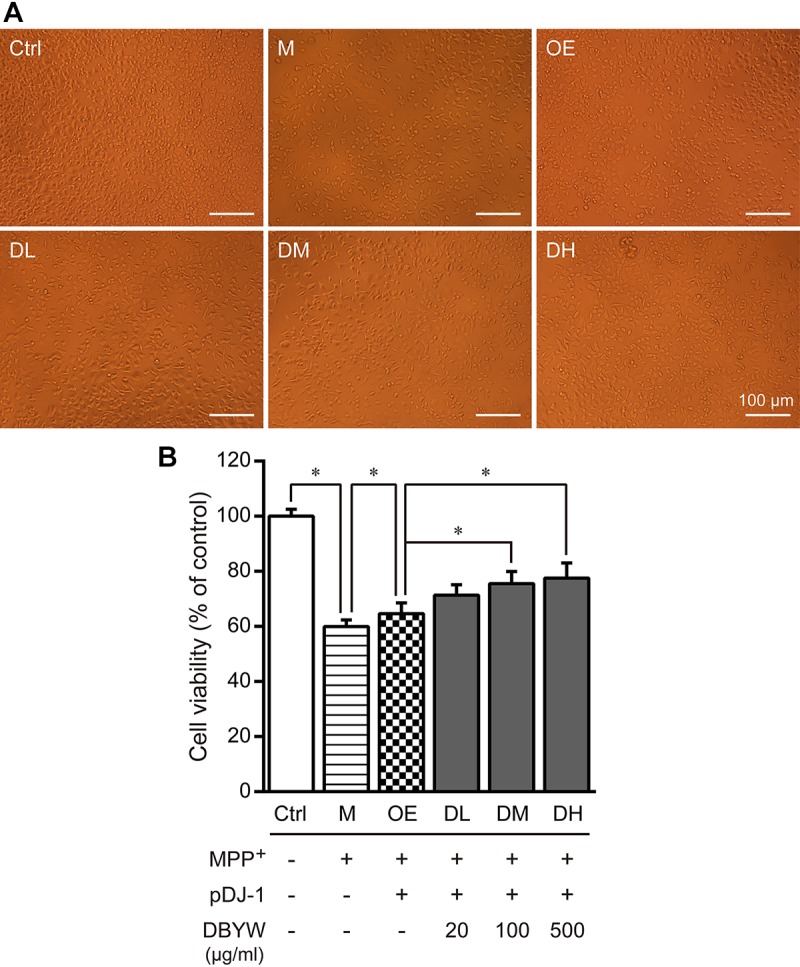
Cell viability detection. **(A)** Representative images showed treatment with MPP^+^ (1 mM) with/without DBYW in the PC-12 cells transfected with pDJ-1. **(B)** Cell viability was detected by CCK-8 assay. Ctrl, the control group; M, the MPP^+^-treated group; OE, the DJ-1 overexpression group; DL/DM/DH, DBYW low/medium/high dose groups; pDJ-1, the plasmid pDJ-1 transfection group. Analysis of variance, *P* < 0.05, *post hoc* ^∗^*P* < 0.05 versus compared group.

The cells were exposed to MPP^+^ (1 mM) with/without different doses of DBYW, respectively. As illustrated in **Figures 3A,B**, MPP^+^ significantly inhibited the cell viability (*P* < 0.05). However, cytotoxic effect of MPP^+^ was ameliorated in PC-12 cells transfected with pDJ-1. Moreover, this effect was promoted by DBYW dose-dependently (*P* < 0.05; **Figures 3A,B**).

### DBYW Affects the DJ-1 Expression

**FIGURE 4:**
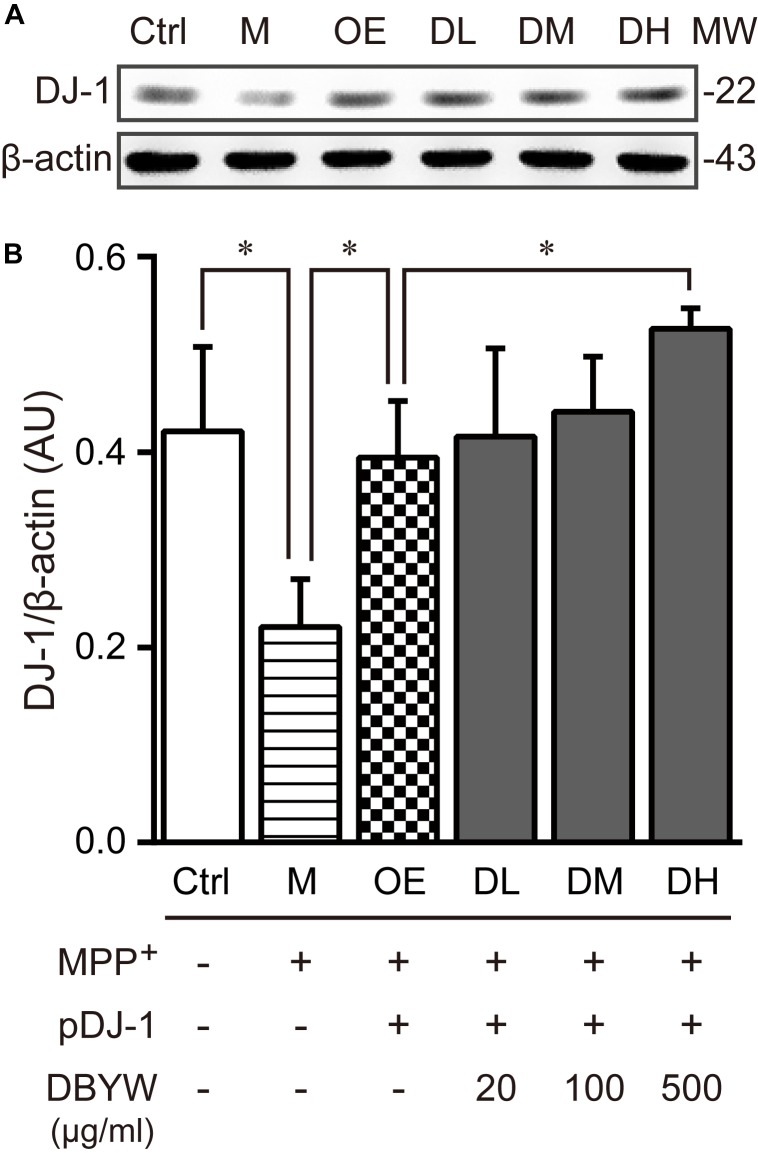
DJ-1 expression detection. **(A)** Representative expressions for DJ-1/internal control are demonstrated. **(B)** The graph displays the analysis from three independent bolts for the expression ratio of DJ-1/beta-actin. AU, arbitrary unit; Ctrl, the control group; M, the MPP^+^-treated group; MW, molecular weight (kDa); OE, the DJ-1 overexpression group; DL/DM/DH, DBYW low/medium/high dose groups; pDJ-1, the plasmid pDJ-1 transfection group. Analysis of variance, *P* < 0.05, *post hoc* ^∗^*P* < 0.05 versus compared group.

To examine the effect of DBYW on the DJ-1 expression, western blot was performed. The results displayed that MPP^+^ (1 mM) treatment decreased the DJ-1 expression (**Figure 4**). The plasmid pDJ-1 transfection inhibited the MPP^+^-induced DJ-1 decreased expression in PC-12 cells. Similarly, DBYW at various concentrations attenuated the MPP^+^-induced decrease of DJ-1 in PC-12 cells with pDJ-1 transfected, in comparison with only pDJ-1 transfection (*P* < 0.05; **Figure 4**). The combined results demonstrated that pDJ-1 could overexpress the protein of DJ-1, and DBYW could promote the DJ-1 expression.

### DBYW Ameliorated the Mitochondrial Dysfunction

**FIGURE 5:**
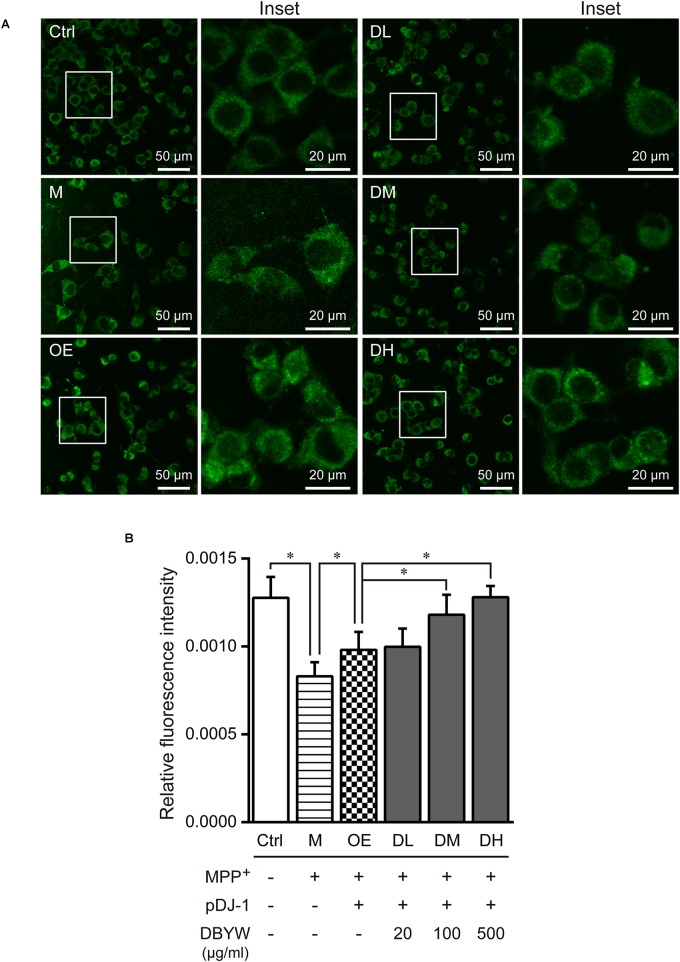
Mitochondrial mass assessment. **(A)** Representative images taken by the confocal microscopy in the different groups. **(B)** Results are the mean ± standard deviation of ten replicates. Ctrl, the control group; M, the MPP^+^-treated group; OE, the DJ-1 overexpression group; DL/DM/DH, DBYW low/medium/high dose groups; pDJ-1, the plasmid pDJ-1 transfection group. Analysis of variance, *P* < 0.05, *post hoc* ^∗^*P* < 0.05 versus compared group.

Confocal fluorescence images evidenced that treatment with MPP^+^ (1 mM) has decreased mitochondrial mass significantly, while pDJ-1 transfection prevented the loss of mitochondrial mass (*P* < 0.05; **Figure 5**). Moreover, different doses of DBYW promoted the mitochondrial mass dose-dependently in the PC-12 cells transfected with pDJ-1, compared to the cells only transfected with pDJ-1 (*P* < 0.05; **Figure 5**).

**FIGURE 6:**
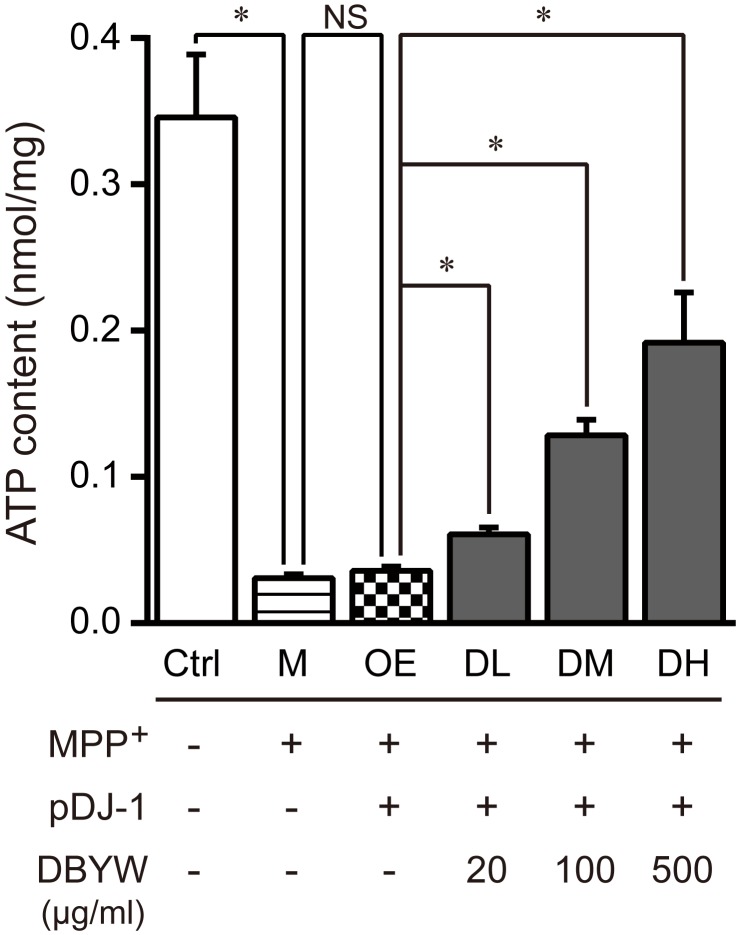
Total ATP content detection. Ctrl, the control group; M, the MPP^+^-treated group; OE, the DJ-1 overexpression group; DL/DM/DH, DBYW low/medium/high dose groups; pDJ-1, the plasmid pDJ-1 transfection group. Analysis of variance, *P* < 0.05, *post hoc* ^∗^*P* < 0.05 versus compared group.

Subsequently, we measured changes in the total ATP content. The data displayed that MPP^+^ (1 mM) treatment significantly reduced the level of total ATP. Similarly, pDJ-1 transfection reversed the reduction in MPP^+^-treated PC-12 cells. Additionally, DBYW at different doses considerably increased total ATP content in a dose-dependent manner in the PC-12 cells transfected with pDJ-1, compared to the cells only transfected with pDJ-1 (*P* < 0.05; **Figure 6**).

### Effect of DBYW on the PI3K/Akt Signaling

**FIGURE 7:**
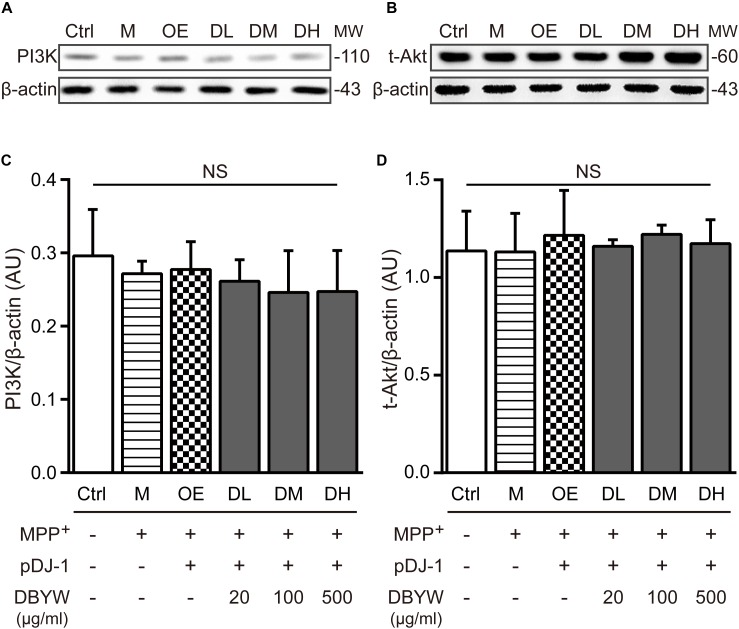
PI3K and total Akt expressions detection. **(A,B)** Representative expressions for PI3K, total Akt, and beta-actin are demonstrated. **(C,D)** The graph shows the analysis in from three independent blots for the expression ratios of PI3K or total Akt, normalized to beta-actin. AU, arbitrary unit; Ctrl, the control group; M, the MPP^+^-treated group; MW, molecular weight (kDa); OE, the DJ-1 overexpression group; DL/DM/DH, DBYW low/medium/high dose groups; pDJ-1, the plasmid pDJ-1 transfection group; NS, not significant statistically.

Western blot results showed that expressions of PI3K or Akt were not affected by MPP^+^ treatment or transfection with pDJ-1. Additionally, DBYW at different doses (20, 100, and 500 μg/ml) could not statistically change the expressions of PI3K or total Akt in the transfected PC-12 cells (*P* > 0.05; **Figure 7**), suggesting that DJ-1 or DBYW could not affect the PI3K and total Akt expressions in MPP^+^-treated PC-12 cells.

**FIGURE 8:**
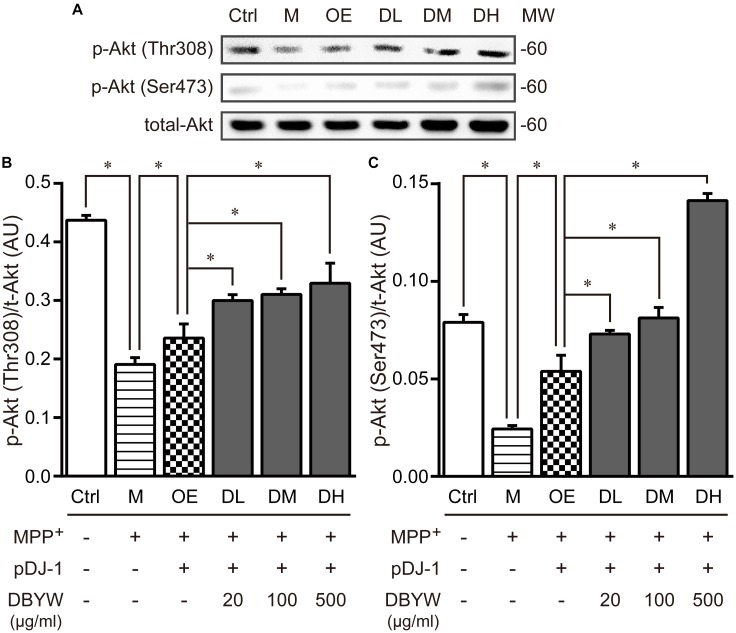
Akt phosphorylation detection. **(A)** Representative expressions for p-Akt^Thr308^, p-Akt^Ser473^, and total Akt are demonstrated. **(B,C)** The graph demonstrates the analysis from three independent blots for the expression ratios of p-Akt^Thr308^ or p-Akt^Ser473^, normalized to total Akt. AU, arbitrary unit; Ctrl, the control group; M, the MPP^+^-treated group; MW, molecular weight (kDa); OE, the DJ-1 overexpression group; DL/DM/DH, DBYW low/medium/high dose groups; pDJ-1, the plasmid pDJ-1 transfection group. Analysis of variance, *P* < 0.05, *post hoc* ^∗^*P* < 0.05 versus compared group.

Subsequently, threonine 308 (Thr308) and serine 473 (Ser473), two residues of Akt phosphorylation (Sarbassov et al., 2005), were investigated by western blot. MPP^+^ (1 mM) treatment significantly decreased the expression of Akt phosphorylation on these two residuses, while pDJ-1 transfection reversed the decrease. Furthermore, different doses of DBYW enhanced the Akt phosphorylation on these two residues dose-dependently, respectively, in the PC-12 cells transfected with pDJ-1 (*P* < 0.05; **Figure 8**), compared to the cells only transfected with pDJ-1. The results displayed that DJ-1 could augment the Akt phosphorylation; and the treatment with DBYW enhanced the effects.

## Discussion

In our previous research, we exposed PC-12 cells to various doses of MPP^+^ for different time periods, respectively. We found a significant loss of PC-12 cells treated with 1 mM MPP^+^ for 48 h. Therefore, we used this condition for PC-12 cells in present research. Additionally, PC-12 cells have been widely served as a cellular model system for investigating PD (Hatanaka, 1981; Westerink and Ewing, 2008), because they have the enzymes for dopamine synthesis, metabolism and transportation (Hatanaka, 1981; Tuler et al., 1989). Our recent research demonstrated that DJ-1 could protect the mitochondria through enhancing the phosphorylation of Akt in MPP^+^-untreated PC-12 cells (Zhang et al., 2016b).

Traditional Chinese medicine formulas or other natural medicines are complex mixtures of many chemical compounds that have diverse pharmacological properties (Zhang Y. et al., 2014). *Anemarrhena asphodeloides* Bge., native to China, Korea, and Mongolia (Wang et al., 2014), is one component of DBYW. Mangiferin is a natural C-glucoside xanthone commonly encountered in *Anemarrhena asphodeloides* Bge. (Vyas et al., 2012). Mangiferin also increases the superoxide dismutase activity and glutathione levels, and prevents depletion of dopamine and its metabolites (3-methoxy-4-hydroxy-phenylacetic acid and homovanillic acid) in the striatum of MPTP-induced mice (Kavitha et al., 2013; Feng et al., 2014). Catalpol, an ingredient abundant in the *Radix Rehmanniae* Praeparata, exerts protective effects on PC-12 cells injured by L-glutamate and Aβ_25-35_ (Wang et al., 2008). In addition, catalpol reverses intracellular calcium level, mitochondrial membrane potential, and reactive oxygen species (ROS) accumulation in MPTP-treated mesencephalic neuron-astrocyte cultures and inhibits the activity of monoamine oxidase B in MPP^+^-treated astrocytes (Bi et al., 2008). The antioxidant α-tocopherol (vitamin E) also significantly increases the synthesis rate and the levels of monoaminergic neurotransmitters in the hippocampus and striatum, brain regions involved in memory processing and motor coordination (Ramis et al., 2016). Additionally, the aqueous extract of *Harpagophytum procumbens* could reduce amyloid β-peptide stimulation of malondialdehyde and 3-hydroxykynurenine and blunt the decrease of dopamine, norepinephrine, and serotonin, in the cortex (Ferrante et al., 2017). Catalpol protects dopaminergic neurons against lipopolysaccharide-induced neurotoxicity dose-dependently, through reducing the release of ROS, nitric oxide, and tumor necrosis factor-α, and attenuating the expression of inducible nitric-oxide synthase in mesencephalic neuron-glia cultures (Tian et al., 2006). Moreover, catalpol could protect mitochondrial function through inhibiting ROS production and nitric oxide synthase activity, increasing activities of mitochondrial complexes and level of mitochondrial membrane potential in the cortex and hippocampus mitochondria of D-galactose injected mouse (Zhang et al., 2010). Furthermore, catalpol improves the locomotor ability dose-dependently and raises the TH neuron number in SN, the density of striatal dopamine transporter, and the protein level of striatal glial cell derived neurotrophic factor in MPTP-treated mice (Xu et al., 2010). Catalpol also attenuates chronic cerebral hypoperfusion-induced white matter lesions by promoting oligodendrocyte survival and oligodendrocyte progenitor differentiation through the Akt signaling in Wistar rats (Cai et al., 2014). Additionally, tetrahydroberberine, an alkaloid isolated from *Phellodendron chinense* Schneid., protects neurons against degeneration through blocking neuronal ATP-sensitive potassium channels in SN of rat (Wu et al., 2010). The extracted decoction of Plastrum Testudinis (Tortoise shell), another component of DBYW, could substantially reduce the rotational behavior (Li et al., 2004; Deng et al., 2008) and increase the TH-positive neurons in the compact zone of substantia nigra (Deng et al., 2008), and also increase the levels of dopamine, 3,4-dihydroxyphenylacetic acid (DOPAC), and homovanillic acid (HVA) in the striatum of the PD model rats (Li et al., 2004). In addition, treatment with the combination of Plastrum Testudinis and β-asarone could improve the behavior of PD rats and increased TH-positive neurons, while decrease α-synuclein level in the corpus striatum (Zhang S. et al., 2014). Additionally, Plastrum Testudinis is one of the main components of Gui-Ling-Pa-An Capsule (GLPAC), a TCM formula that is used in the treatment for PD clinically. A multicenter, randomized, double-blind, controlled clinical trial has demonstrated that GLPAC shows obvious effects in improving the motor syndrome and quality of life of PD patients, and also reduces the required dosage of levodopa (Zhao et al., 2009).

Akt signaling defection has partly involved in the neurodegenerative progression of Alzheimer’s and Huntington’s diseases (Colin et al., 2005; Griffin et al., 2005). In addition, Akt signaling has a crucial role in mediating the dopaminergic receptor (Beaulieu et al., 2007) and redistributing the dopaminergic transporters (Garcia, 2005). Moreover, some PD treatment drugs (e.g., bromocriptine and ropinirole) that targeting the dopaminergic system had demonstrated the neuroprotective effects via Akt signaling (Lim et al., 2008; Nair and Olanow, 2008). Akt activation could induce various biological responses, such as insulin metabolic function, oncogenic signal transduction, higher brain function linked to cognition (Franke, 2008). Phosphorylation is a crucial modulatory mechanism that occurs both in prokaryotic and eukaryotic organisms (Barford et al., 1998), and the significant post-translational determinant of Akt activity (Sarbassov et al., 2005). Rasagiline, a selective monoamine oxidase B inhibitor, could up-regulate the Akt phosphorylation on Ser473 in midbrain dopamine neurons in MPTP-induced mice (Sagi et al., 2007). Furthermore, both gain-of-function and loss-of-function experiments have demonstrated that DJ-1 promotes cell survival *via* Akt phosphorylation on Ser473 in *Drosophila* (Kim et al., 2005). Additionally, using the DJ-1 null mouse model of PD, reduced Akt phosphorylation on Ser473 is associated with gradual loss of neurons in SN (Aleyasin et al., 2010). Further studies *in vivo* should be performed to better understand these *in vitro* findings.

In summary, these results demonstrate that DJ-1 could ameliorate the mitochondrial dysfunction at least through medicating the Akt phosphorylation in the rat adrenal pheochromocytoma PC-12 cells treated with MPP^+^. Additionally, our findings also suggest that DBYW promotes the ameliorative effect of DJ-1 in the MPP^+^-treated PC-12 cells.

## Author Contributions

YZ and HMS conceptualized the study. YZ, XGG, ZZW, and HMS analyzed the data. YZ, XGG, ZYG, JHH, YYW, and WDF performed the experiments. YZ drafted and finalized the paper. ZZW, HMS, LL, PL, and NHC were the contributors in writing and revising the manuscript.

## Conflict of Interest Statement

The authors declare that the research was conducted in the absence of any commercial or financial relationships that could be construed as a potential conflict of interest.

## Footnotes

1 http://www.nifdc.org.cn
